# Supplementary material for: A transgenic zebrafish for spatiotemporal optogenetic activation of FGF/ERK signaling
Source: Biol Open. 2026 Sep 9;15(9):bio062695. doi: 10.1242/bio.062695 (PMC13602372; doi:10.1242/bio.062695)
Supplement: Supplementary file 1 [file biolopen-15-062695-s1.pdf]

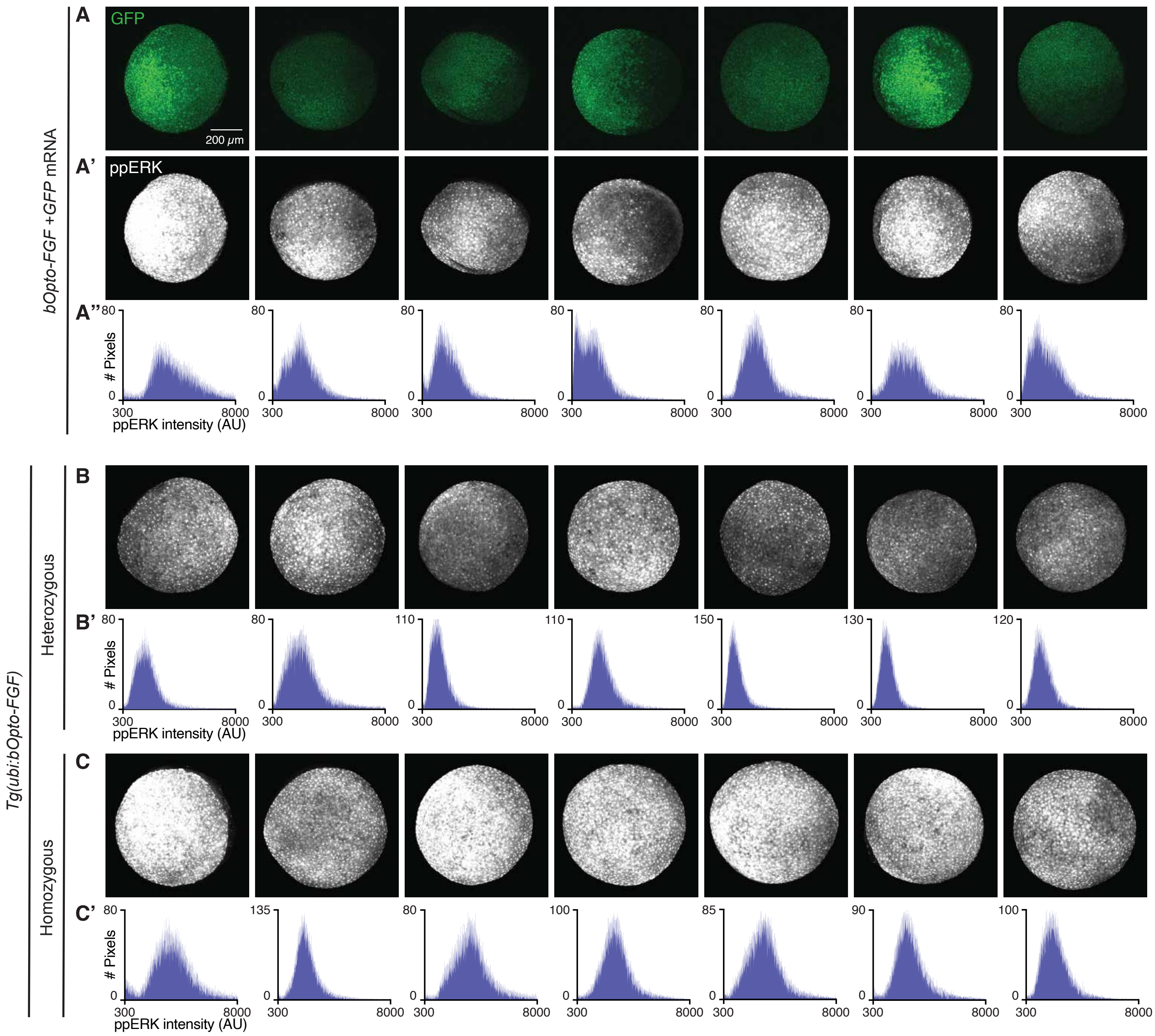

Fig. S1. Spatial uniformity of optogenetic FGF/ERK signaling activation. *Tg(ubi:bOpto-FGF)* and *bOpto-FGF* + *GFP* mRNA-injected embryos were exposed to 455 nm light for 30 min beginning at shield stage. Embryos were fixed along with unexposed controls. HCR-IF staining for ppERK was used to quantify FGF/ERK signaling. **A-A')** GFP signal (A) and ppERK signal (A') from light exposed mRNA-inject-ed embryos from one replicate. **B,C)** ppERK signal from light exposed heterozygous (B) and homozygous (C) embryos from one replicate. Scale bar = 200 μm. **A''**,**B'**,**C'**) Histograms of ppERK pixel intensities from above images. Note that y-axes may differ for display purposes.

Biology Open • Supplementary information

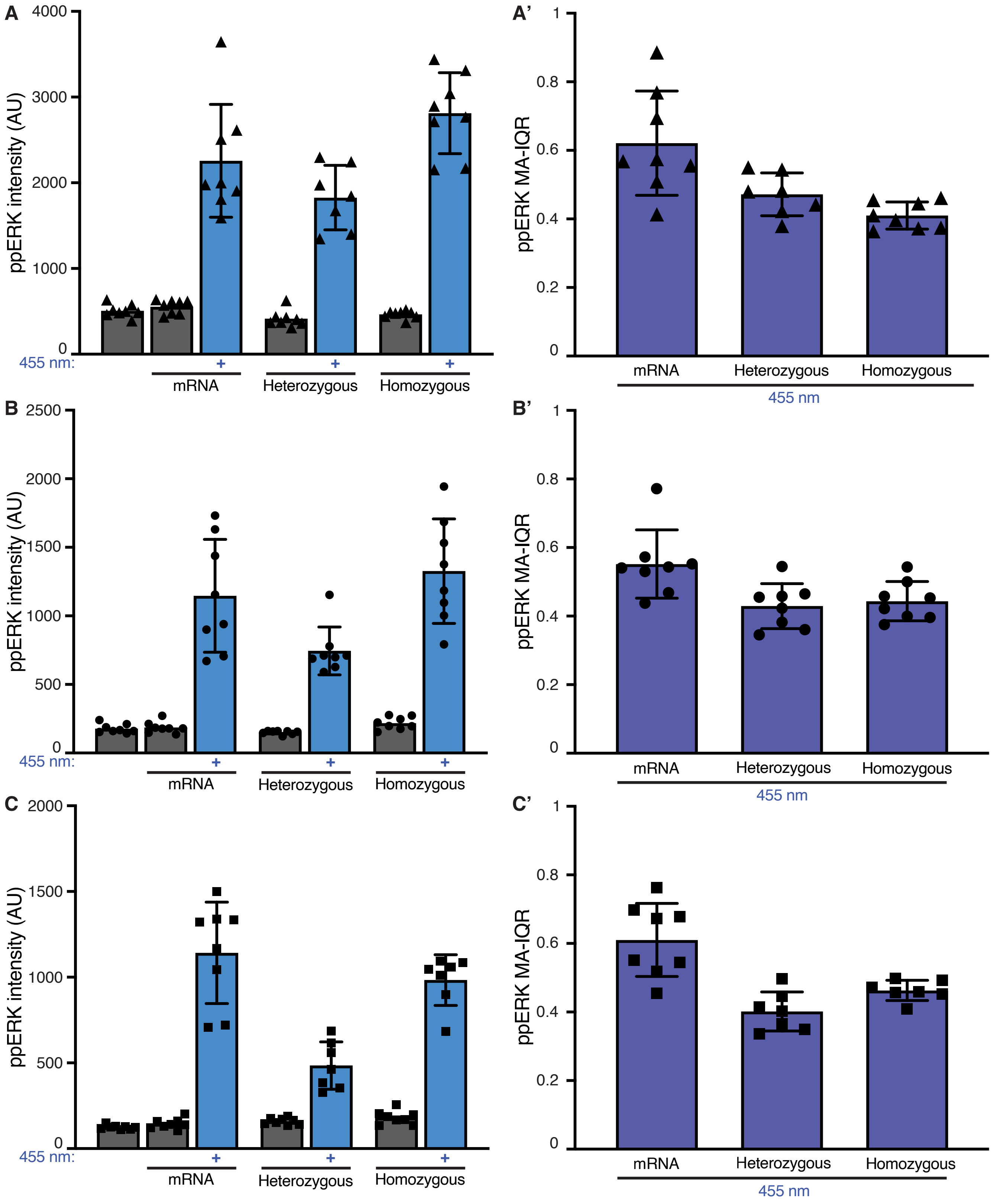

Fig. S2. Optogenetic FGF/ERK activation replicates. *Tg(ubi:bOpto-FGF)* and *bOpto-FGF* + *GFP* mRNA-injected embryos were exposed to 455 nm light for 30 min beginning at shield stage. Embryos were fixed along with unexposed controls. mRNA replicates also included uninjected, unexposed controls. HCR-IF staining for ppERK was used to quantify FGF/ERK signaling. **A,B,C)** Raw ppERK intensities from three replicates. Each data point shows the average ppERK intensity from one embryo. **A',B',C')** Corresponding ppERK mean-adjusted interquartile ranges (MA-IQR) from the three replicates shown in A, B, and C. Each data point shows the MA-IQR from one embryo. For all graphs, the symbol used for each replicate is consistent with Fig. 2, and error bars show SD.

Biology Open • Supplementary information

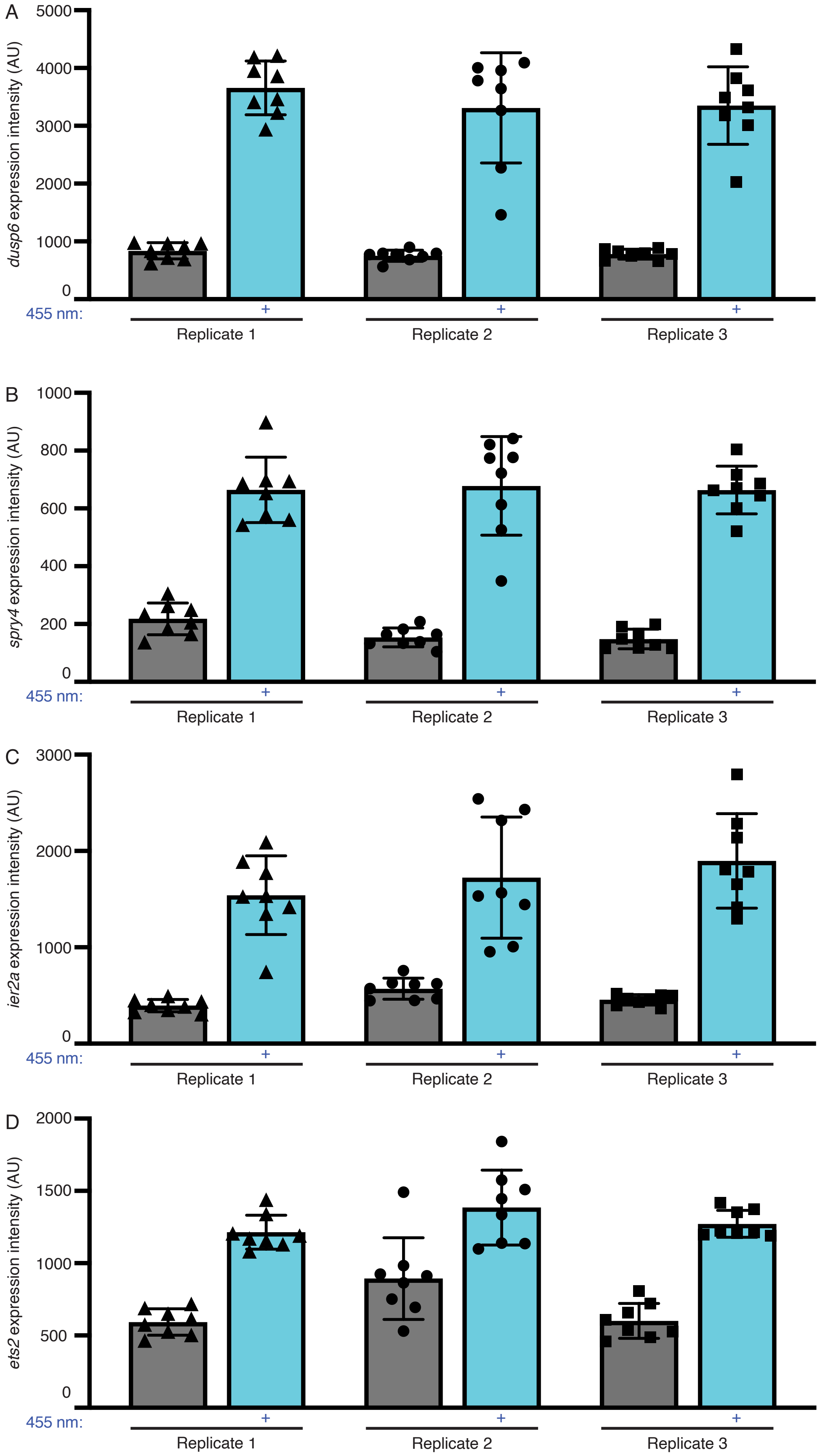

Fig. S3. Light-mediated activation of FGF target gene expression. *Tg(ubi:bOpto-FGF*) heterozy-gous embryos were exposed to 455 nm light for 45 min beginning at 50% epiboly. Exposed embryos were fixed along with unexposed controls and HCR-FISH was performed for the indicated genes. **A-D)** Raw HCR-FISH intensity data for each gene from three replicates. Each data point shows the average intensity from one embryo. The symbol used for each replicate is consistent with Fig. 2. Error bars show SD.

Biology Open • Supplementary information

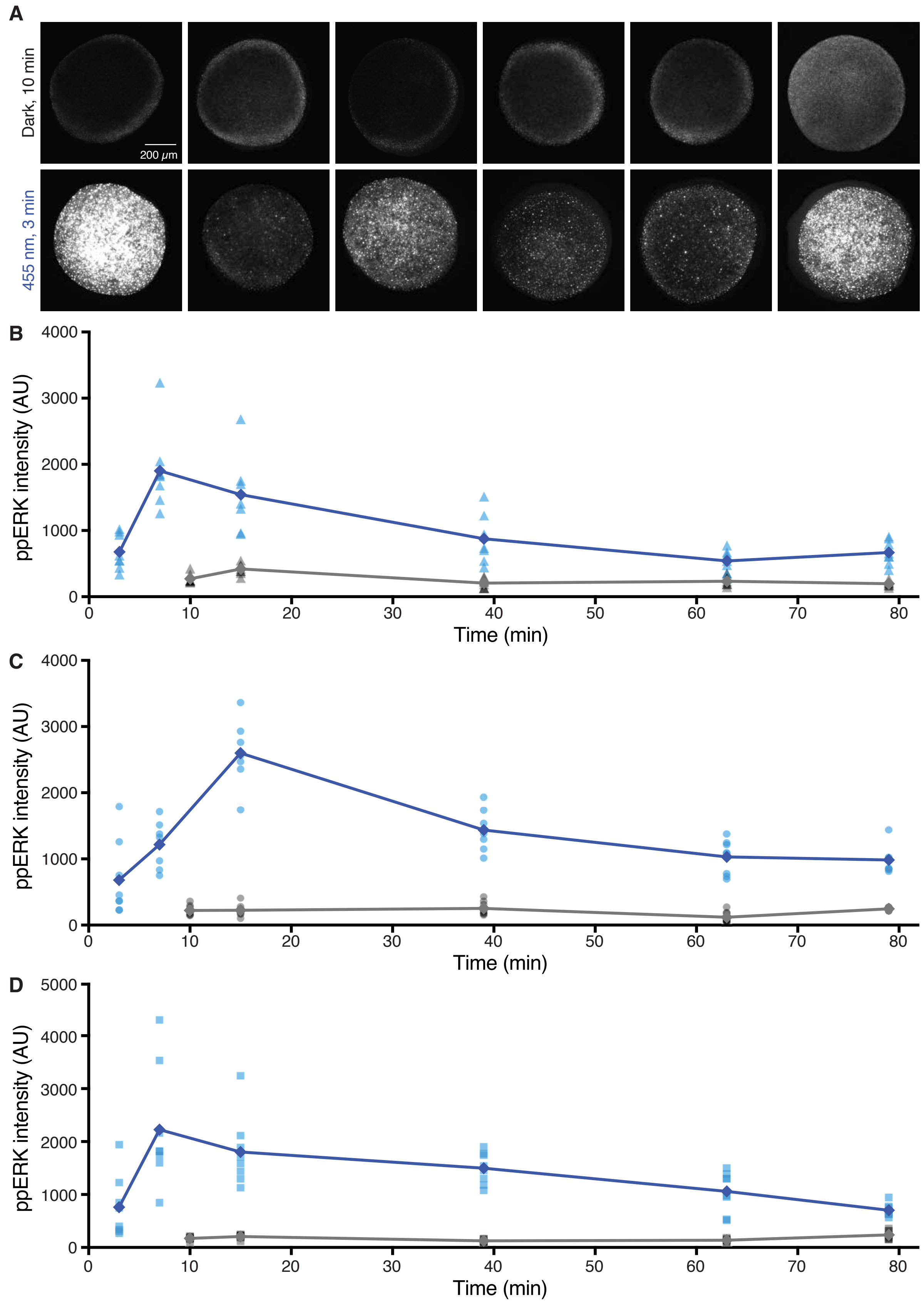

Fig. S4. Kinetics independent replicates & representative 3 min images. Heterozygous *Tg(ubi:bOpto-FGF)* embryos were exposed to 455 nm light beginning at 30% epiboly. Embryos were fixed along with time-matched unexposed controls at the indicated durations. HCR-IF ppERK staining was used to assess FGF/ERK activity. **A)** Representative images of embryos exposed to blue light and fixed at 3 min (bottom row) and dark controls fixed at 10 min (top row). Scale bar = 200 μm. **B-D)** Raw ppERK intensity data from three replicates. Each data point (trian-gle, circle, or square) shows the average ppERK intensity from one embryo. The symbol used for each replicate is consistent with Fig. 3. Diamonds represent averaged data.

Biology Open • Supplementary information

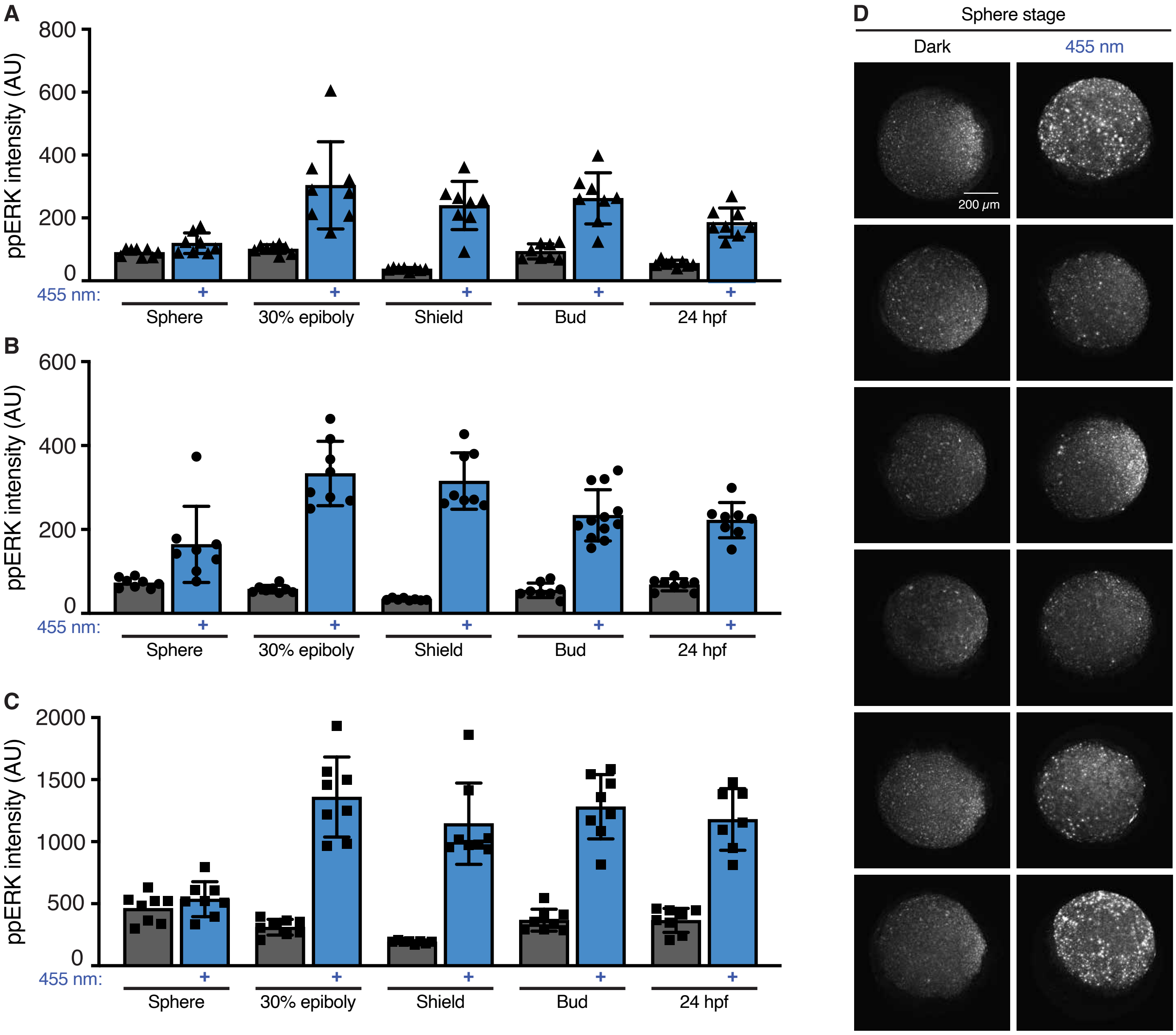

Fig. S5. Timecourse replicates & representative sphere stage images. Heterozygous *Tg(ubi:bOpto-FGF)* embryos were exposed to 455 nm light beginning at the indicated stages. Embryos were fixed after 30 min along with stage-matched unexposed controls. HCR-IF for ppERK was used to detect FGF/ERK signaling activity. **A-C)** Raw ppERK intensity data from three replicates. Each data point shows the average ppERK intensity from one embryo. The symbol used for each replicate is consistent with Fig. 4. Error bars show SD. **D)** Representative images of blue light-exposed sphere-stage heterozygous embryos (right) and unexposed controls (left). Scale bar = 200 μm.

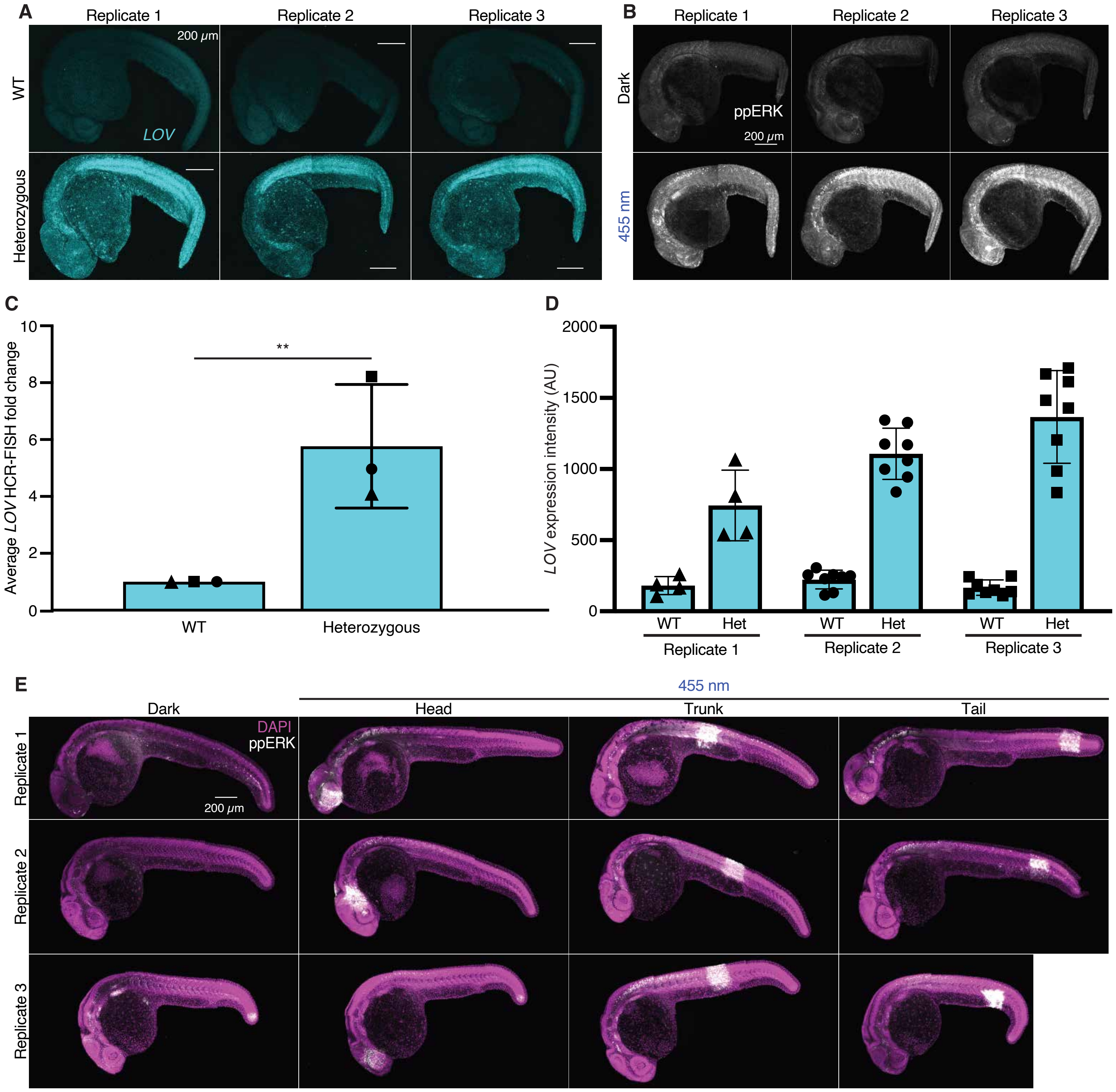

Fig. S6. Distribution of *bOpto-FGF* mRNA & FGF/ERK activation after uniform or localized light exposure at 1 dpf. A, C and D: Dark-reared wild-type (top row) and *Tg(ubi:bOpto-FGF)* heterozygous embryos (bottom row) were fixed at 24 hpf. HCR-FISH was used to detect *bOpto-FGF* mRNA using probes targeting the *LOV* domain. **A)** Representative images from three replicates are shown. **B)** *Tg(ubi:bOpto-FGF)* heterozygous embryos were raised in the dark until 24 hpf, then either exposed to 455 nm light for 30 min (bottom row) or kept in the dark (top row), then stained for ppERK. Representative images from three replicates shown. **C)** Average intensity fold changes for three replicates of HCR-FISH for *LOV* mRNA. **D)** Raw intensity data from three *LOV* HCR-FISH replicates. Each data point shows the average intensity from one embryo. The symbol used for each replicate is consistent with **C**. Error bars show SD. **E)** *Tg(ubi:bOpto-FGF)* heterozygous embryos were raised in the dark until 1 dpf. Embryos were then exposed to a 170 x 170 μm square of 455 nm light for 1 min using a digital

Biology Open • Supplementary information

Table S1. List of fixed effects and post-hoc analyses used

Analysis	Figure	Fixed effects + p-values	Post-hoc analysis
1 – ppERK intensity comparisons	2C	1. Exposure (dark or blue) ($p < 0.0001$)<br>2. Embryo type (Injected, het, or hom) ($p = .00036$)<br>3. Exposure*embryo type ($p = .00036$)	Tukey's Honest Significant Difference (HSD)
2 – Mean-adjusted interquartile range	2D	1. Embryo type ($p < 0.0001$)	Tukey's HSD
3 – Gene expression intensity comparisons	2F	1. Exposure (dark or blue) ($p < 0.0001$ for both co-stained sets)<br>2. Gene (p = 0.037 for dusp6/spry4 and p < 0.0001 for ets2 and ier2a)<br>3. Exposure*gene (p = 0.037 for dusp6/spry4 and p < 0.0001 for ets2 and ier2a)	Tukey's HSD
4 – Duration comparisons	3B	1. Exposure ($p < 0.0001$)<br>2. Exposure duration ($p < 0.0001$)<br>3. Exposure*exposure duration ($p < 0.0001$)	Student's t-test with a Bonferroni correction
5 – Stage comparisons	4B	1. Exposure ($p < 0.0001$)<br>2. Stage ($p < 0.0001$)<br>3. Exposure*Stage ($p < 0.0001$)	Tukey's HSD
6 – LOV mRNA quantification	S6D	1. WT vs. Het (p = 0.0078)	N/A (only one comparison made)

Table S2. Corrected P-values from intensity analysis in Fig. 2C.

(Analysis 1: Linear mixed-effects model with Tukey's HSD to correct for multiple comparisons)

Comparison	P-value
Injected + Dark vs. Injected + Blue	P <0.0001
Heterozygous + Dark vs. Heterozygous + Blue	P <0.0001
Homozygous + Dark vs. Homozygous + Blue	P <0.0001
Injected + Blue vs. Heterozygous + Blue	P <0.0001
Injected + Blue vs. Homozygous + Blue	P = 0.9986
Heterozygous + Blue vs. Homozygous + Blue	P <0.0001

Biology Open • Supplementary information

Table S3. Corrected P-values from mean-adjusted interquartile range analysis in Fig. 2D.

(Analysis 2: Linear mixed-effects model with Tukey's HSD to correct for multiple comparisons)

**Comparison**	**P-value**
Injected + Blue vs. Heterozygous + Blue	P <0.0001
Injected + Blue vs. Homozygous + Blue	P <0.0001
Heterozygous + Blue vs. Homozygous + Blue	P = 0.9852

Table S4. Corrected P-values from HCR-FISH in Fig. 2F.

(Analysis 3: Linear mixed-effects model with Tukey's HSD to correct for multiple comparisons)

**Comparison**	**P-value**
dusp6: Dark vs. Blue	P <0.0001
spry4: Dark vs. Blue	P <0.0001
ets2: Dark vs. Blue	P <0.0001
ier2a: Dark vs. Blue	P <0.0001

Table S5A. Dark vs. blue corrected p-values for kinetics analysis in Fig. 3B.

(Analysis 4: Linear mixed-effects model with Bonferroni corrections following Student's t-tests)

**Comparison**	**P-value**
10 min + Dark vs. 3 min + Blue	P = 0.0155
10 min + Dark vs. 7 min + Blue	P <0.0001
15 min + Dark vs. 15 min + Blue	P <0.0001
39 min + Dark vs. 39 min + Blue	P <0.0001
63 min + Dark vs. 63 min + Blue	P <0.0001
79 min + Dark vs. 79 min + Blue	P = 0.0081

Table S5B. Time point comparison corrected p-values for analysis in Fig. 3B.

(Analysis 4: Linear mixed-effects model with Bonferroni corrections following Student's t-tests)

**Comparison**	**7 min**	**15 min**	**39 min**	**63 min**	**79 min**
3 min	P <0.0001	P <0.0001	P <0.0001	P = 0.0006	P = 1.0
7 min	-	P = 1.0	P = 1.0	P = 0.0231	P <0.0001
15 min	-	-	P = 1.0	P = 0.1050	P <0.0001
39 min	-	-	-	P = 1.0	P <0.0001
63 min	-	-	-	-	P = 0.0013

Biology Open • Supplementary information

Table S6A. Dark vs. blue corrected p-values for stage analysis in Fig. 4B.

(Analysis 5: Linear mixed-effects model with Tukey's HSD to correct for multiple comparisons)

**Comparison**	**P-value**
Sphere + Dark vs. Sphere + Blue	P = 0.5748
30% epiboly + Dark vs. 30% epiboly + Blue	P <0.0001
Shield + Dark vs. Shield + Blue	P <0.0001
Bud + Dark vs. Bud + Blue	P <0.0001
24 hpf + Dark vs. 24 hpf + Blue	P <0.0001

Table S6B. Stage comparison corrected p-values for analysis in Fig. 4B.

(Analysis 5: Linear mixed-effects model with Tukey's HSD to correct for multiple comparisons)

**Comparison**	**30% Epiboly**	**Shield**	**Bud**	**24hpf**
Sphere	P <0.0001	P <0.0001	P <0.0001	P <0.0001
30% Epiboly	-	P <0.0001	P = 0.0332	P = 0.0058
Shield	-	-	P <0.0001	P <0.0001
Bud	-	-	-	P = 0.9999

Dataset 1. HCR-FISH probe sequences

Available for download at
https://journals.biologists.com/bio/article-lookup/doi/10.1242/bio.062695#supplementary-data
