# Supplementary material for: A transgenic zebrafish for spatiotemporal optogenetic activation of FGF/ERK signaling
Source: Biol Open. 2026 Sep 9;15(9):bio062695. doi: 10.1242/bio.062695 (PMC13602372; doi:10.1242/bio.062695)
Supplement: Supplementary file 2 [file biolopen-15-062695_review_history.pdf]

A transgenic zebrafish for spatiotemporal optogenetic activation of FGF/ERK signaling

William K. Anderson, Leanne E. Iannucci, Ninet Sinaii, Julia Porcino and Katherine W. Rogers

10.1242/bio.062695

Editor: Daniel Gorelick

Review timeline

Original submission:	12 May 2026
Editorial decision:	19 May 2026
First revision received:	13 August 2026
Accepted:	16 August 2026

Original submission

First decision letter

MS ID#: bio.062695

MS Title: A transgenic zebrafish for spatiotemporal optogenetic activation of FGF/ERK signaling

Authors: William K. Anderson, Leanne E. Iannucci, Ninet Sinaii, Julia Porcino and Katherine W. Rogers

Thank you for submitting your manuscript to Biology Open, and participating in our Fast & Fair peer review process, where we provide authors a decision, with reviews, within 7 working days of submission. We can do this because we pre-contract with and pay peer reviewers.

I have now reached a decision on the above manuscript.

The reviewer reports are shown at the bottom of this email.

As you will see, the reviewers gave favourable reports, but raised some points that will require amendments to your manuscript. I hope that you will be able to carry these out, because we would like to be able to accept your paper.

At this stage, we also ask you to ensure your manuscript complies with our formatting guidelines – please see our manuscript preparation guidelines for details. Provided you are able to fully address the referees' comments, we are positive about publication of your paper (we accept over 95% of revision submissions) and therefore hope you won't mind any extra work involved in reformatting your manuscript at this point.

Please upload both a 'clean' version of your Word file, along with a highlighted version clearly showing where you have made changes in the revised manuscript. Please avoid using 'Track changes' in Word files as these are lost in PDF conversion.

I should be grateful if you would also provide a point-by-point response detailing how you have dealt with the points raised by the reviewers in the 'Response to Reviewers' box. Please attend to

© 2026. Published by The Company of Biologists under the terms of the Creative Commons Attribution License (https://creativecommons.org/licenses/by/4.0/).

all of the reviewers' comments. If you do not agree with any of their criticisms or suggestions please explain clearly why this is so.

Reviewer 1

Comments for the author

This is an excellent and useful Methods & Techniques manuscript describing the establishment and partial characterisation of a novel transgenic zebrafish line for optogenetic activation of FGF/ERK signalling. The study is well written, technically careful, and highly relevant to the zebrafish and signalling communities. The authors provide a convincing rationale for moving from mRNA injection to a stable transgenic line and show robust light-dependent ERK activation, improved spatial uniformity compared with injected embryos, and responsiveness across relevant developmental stages. Overall, the manuscript is a good fit for Biology Open and represents a valuable resource.

Major criticisms

1. The ppERK antibody staining is convincing and appropriate as a proximal readout of ERK activation. However, the manuscript would be strengthened by a clearer demonstration that light activation of the transgene leads to a broader FGF/ERK pathway response. At present, the conclusions rest largely on ppERK levels, and an additional read-out for FGF/ERK activation would be beneficial.
2. A major attraction of such an optogenetic tool is the possibility to activate signalling in a defined region while leaving neighbouring tissue unactivated within the same embryo. The manuscript would benefit from an example for spatially restricted activation.
3. The authors state that the line is responsive from around 30% epiboly through at least 24 hpf. This is useful, but the wording could be misread as suggesting sustained activation across this full developmental period. Since broad FGF/ERK overactivation during early zebrafish development would be expected to cause strong morphological defects, the authors should clarify the exact activation regimes used and better explain how pathway activation relates to expected developmental phenotypes, survival, and morphology.
4. The line uses the ubi promoter, which is expected to drive broad expression, and the functional data support widespread light responsiveness under the tested conditions. However, direct expression of the optogenetic construct across all tissues and stages is not extensively characterised in this manuscript and should be reworded.

Minor criticisms

1. The introduction could better reflect established FGF/ERK biology in neural patterning, given its pivotal role in MHB emergence
2. The data suggest robust responsiveness from 30% epiboly onwards, while sphere-stage responses appear variable or not significant overall. The wording should reflect this distinction more precisely.
3. The analysis appears careful, but the manuscript would benefit from a clearer description of how ppERK signal was quantified and how spatial uniformity was assessed. In particular, the MA-IQR metric should be introduced in a way that is accessible to non-specialist readers.
4. The finding that the fish can be maintained under the authors' standard facility lighting is useful. However, because lighting conditions vary between facilities, the conclusions and recommendations should remain appropriately cautious.
5. The possibility that this line may be useful beyond 24 hpf or in adults is interesting, but this remains speculative based on the present data. The manuscript should clearly separate demonstrated utility from future potential.

This is a strong, well-executed and valuable resource paper. The manuscript is clearly suitable for Biology Open as a Methods & Techniques article. The main conclusions are supported, but several points require clarification, particularly the breadth of pathway validation, spatial activation claims, and interpretation of developmental consequences after activation.

© 2026. Published by The Company of Biologists under the terms of the Creative Commons Attribution License (https://creativecommons.org/licenses/by/4.0/).

Reviewer 2

Comments for the author

This manuscript describes the generation and characterization of Tg(ubi:bOpto-FGF), a transgenic zebrafish line that ubiquitously expresses the bOpto-FGF optogenetic tool for blue light-inducible activation of FGF/ERK signaling. The authors demonstrate robust, spatially uniform ERK phosphorylation upon blue light exposure in both heterozygous and homozygous embryos, characterize light responsiveness across developmental stages from late blastula through 24 hpf, and show that ectopic signaling is induced rapidly (within 3 minutes) and sustained for at least 75 minutes under continuous illumination. This is a useful methodological contribution to the zebrafish optogenetics toolkit.

Some points to consider for a revision:
The MATLAB-based quantification pipeline is referenced from a different paper (Iannucci et al., 2026), which is currently a preprint. The authors should either provide the code as supplementary material, deposit it in a public repository.

The claim of "ubiquitous" expression, central to the line's utility, is based on the known behavior of the ubi promoter as characterized in prior work (Mosimann et al., 2011). No direct evidence of uniform bOpto-FGF transgene expression across cell types is presented here, for example by anti-HA staining or fluorescent in situ for thetrangene. For a methods paper where this is a primary selling point, at minimum one representative image demonstrating broad expression would be expected including at later stages as this would increase the applicability of the method.

The insertion site of the transgene is unknown. This ideally should be mapped in a primarily method paper presenting the transgenic line as main tool.

To avoid possible activation of the construct by ambient light in fish raised for the line maintenance, the author should consider a bipartite system such as UAS/Gal4, Cre/Lox or QF/QUAS. This should be more clearly discussed in the manuscript.

Minor Comments

1. Figure 4A legend notes that some time points were imaged with different settings. This aspect should also be noted in the main results text.
2. The Iannucci et al., 2026 reference is currently a bioRxiv preprint. If it remains unpublished and not peer reviewed at the time of final submission, this should be noted, and key methodological details from that paper that are not reproduced here should be included.

Reviewer's Responses to Questions

Experimental quality
Are experiments performed using appropriate methods that will answer the question (or test the hypothesis or support the observations) posed by the authors? Is the right tool used for the job?
If 'No', please indicate reasons in Comments for Author box below.
Reviewer #1:
- Yes

Reviewer #2:
- Yes

Were the data analyzed using appropriate statistical tests?
If 'No', please indicate reasons in Comments for Author box below.
Reviewer #1:
- Yes

Reviewer #2:

© 2026. Published by The Company of Biologists under the terms of the Creative Commons Attribution License (https://creativecommons.org/licenses/by/4.0/).

- Yes

Reproducibility
Were experiments performed using adequate number of biological replicates?
If 'No', please indicate reasons in Comments for Author box below.
Reviewer #1:

- Yes

Reviewer #2:

- Yes

Is there sufficient raw data to assess the rigor of the analysis?
If 'No', please indicate reasons in Comments for Author box below.
Reviewer #1:

- Yes

Reviewer #2:

- Yes

Does the methods section provide sufficient detail to permit reproducibility?
If 'No', please indicate reasons in Comments for Author box below.
Reviewer #1:

- Yes

Reviewer #2:

- Yes

Completeness
Are the manuscript's conclusions supported by the data?
If 'No', please indicate reasons in Comments for Author box below.
Reviewer #1:

- Yes

Reviewer #2:

- No

Scholarship
Do the authors cite and discuss the merits of data that would argue for and against their conclusion?
If 'No', please indicate reasons in Comments for Author box below.
Reviewer #1:

- Yes

Reviewer #2:

- Yes

Does the manuscript title & abstract accurately reflect the contents of the manuscript, without hyperbole?
If 'No', please indicate reasons in Comments for Author box below.
Reviewer #1:

- Yes

Reviewer #2:

© 2026. Published by The Company of Biologists under the terms of the Creative Commons Attribution License (https://creativecommons.org/licenses/by/4.0/).

- Yes

First revision

Author response to reviewers' comments

We thank the reviewers for their insightful feedback and are grateful for the opportunity to improve our manuscript.

Reviewer 1: This is an excellent and useful Methods & Techniques manuscript describing the establishment and partial characterisation of a novel transgenic zebrafish line for optogenetic activation of FGF/ERK signalling. The study is well written, technically careful, and highly relevant to the zebrafish and signalling communities. The authors provide a convincing rationale for moving from mRNA injection to a stable transgenic line and show robust light-dependent ERK activation, improved spatial uniformity compared with injected embryos, and responsiveness across relevant developmental stages. Overall, the manuscript is a good fit for Biology Open and represents a valuable resource.

We are glad the reviewer agrees that *Tg(ubi:bOpto-FGF)* is a valuable resource.

Major criticisms

1. The ppERK antibody staining is convincing and appropriate as a proximal readout of ERK activation. However, the manuscript would be strengthened by a clearer demonstration that light activation of the transgene leads to a broader FGF/ERK pathway response. At present, the conclusions rest largely on ppERK levels, and an additional read-out for FGF/ERK activation would be beneficial.

As an additional read-out for FGF/ERK signaling activation, we performed hybridization chain reaction fluorescence *in situ* hybridization (HCR-FISH) in heterozygous transgenic embryos exposed to blue light for 45 min starting at 50% epiboly. Using probes against three previously identified FGF target genes (*spry4*, *dusp6*, *ier2a*) and a gene within a family known to be FGF-activated (*ets2*), we found significant upregulation of all four genes in light-exposed embryos compared to dark controls. This supports the idea that light exposure broadly activates the FGF/ERK signaling pathway.

We now include this data in Fig.2, Fig. S3, and lines 220 – 230 in the "changes tracked" document.

2. A major attraction of such an optogenetic tool is the possibility to activate signalling in a defined region while leaving neighbouring tissue unactivated within the same embryo. The manuscript would benefit from an example for spatially restricted activation.

We agree with the reviewer that spatially localized signaling activation is a powerful benefit of optogenetic strategies. In another recently published manuscript we demonstrated spatially localized activation of ERK phosphorylation in mRNA-injected shield stage embryos (Iannucci et al., 2026). To determine whether *Tg(ubi:bOpto-FGF)* also enables spatially restricted activation at later stages, we exposed the head, trunk, or tail of 1 day post-fertilization *Tg(ubi:bOpto-FGF)* heterozygotes to a 170 x 170 μm square of 455 nm light for 1 min using a digital micromirror device. Embryos were immediately fixed and ppERK IF was performed. We observed spatially localized activation of FGF/ERK signaling in the target area in three biological replicates, confirming that spatial control of signaling is also possible with this transgenic.

We now include this data in Fig.5, Fig. S6, and lines 289 – 298 in the "changes tracked" document.

3. The authors state that the line is responsive from around 30% epiboly through at least 24 hpf. This is useful, but the wording could be misread as suggesting sustained activation across this full developmental period. Since broad FGF/ERK overactivation during early zebrafish development

© 2026. Published by The Company of Biologists under the terms of the Creative Commons Attribution License (https://creativecommons.org/licenses/by/4.0/).

would be expected to cause strong morphological defects, the authors should clarify the exact activation regimes used and better explain how pathway activation relates to expected developmental phenotypes, survival, and morphology.

We thank the reviewer for pointing out the potential for confusion. We do indeed observe lysis at 2 dpf when embryos are constantly exposed to light starting at 2 hpf (Fig.1D), consistent with the severe patterning defects that have been observed from long-term ectopic FGF activation (Iannucci et al., 2026). This has been a helpful readout for assessing tool activity. However, we have not assessed the phenotypic outcomes from the shorter exposures used in Fig. 2-5.

We have altered the wording in the manuscript to address the potential for confusion (line numbers refer to "changes tracked" document):

Line 181 - 182: "Although *Tg(ubi:bOpto-FGF)* embryos **exposed to continuous blue light starting at 2 hpf typically** lyse by 2 dpf (Fig. 1D) consistent with ectopic FGF/ERK activation, lysis is a non-specific phenotype."

Line 264 - 268: "To determine whether *Tg(ubi:bOpto-FGF)* can reliably activate FGF/ERK signaling during these developmental stages, we exposed embryos to 455 nm light for 30 minutes starting at five stages: sphere, 30% epiboly, shield, bud, and 24 hpf (Kimmel et al., 1995). Exposed embryos and stage-matched dark controls **were fixed immediately after the 30 minutes** and HCR-IF was performed to quantify changes in ppERK levels"

4. The line uses the ubi promoter, which is expected to drive broad expression, and the functional data support widespread light responsiveness under the tested conditions. However, direct expression of the optogenetic construct across all tissues and stages is not extensively characterised in this manuscript and should be reworded.

We appreciate the note from both reviewers that we have not characterized the spatiotemporal distribution of the *bOpto-FGF* mRNA or protein. We initially focused our analyses on signaling activation, because this is the final "readout" and ultimately the aim of the tool. We therefore assessed the uniformity of signaling activation at shield stage and found results consistent with uniform expression from the ubi promoter (Fig. 2; Fig. S1, S2).

However, to more directly assess the distribution of the bOpto-FGF construct, we first tried to use immunofluorescence staining against the HA tag (Fig. 1B) to detect the bOpto-FGF construct in 24 hpf heterozygous or homozygous transgenic embryos (using wild-type as a control). Unfortunately, we were unable to detect interpretable levels of HA staining.

We then carried out HCR-FISH using probes targeting the *LOV* domain in 24 hpf heterozygous and wild-type embryos. Quantification of HCR-FISH signal showed a significant increase in *LOV* mRNA in the transgenic compared to wild-types. We detected *LOV* mRNA signal throughout transgenic embryos at this stage, with a possible enrichment in the somites. Despite the potentially non-uniform mRNA distribution at 24 hpf, we observe robust activation of ectopic FGF/ERK signaling in non-somite tissues including the head and tail based on ppERK HCR-IF. This is shown both in experiments in which embryos were uniformly light exposed, as well as in our new experiments demonstrating spatially localized signaling activation in the head, trunk, and tail at 1 dpf. This demonstrates that sufficient bOpto-FGF is present in these tissues to support light-mediated ectopic signaling activation.

We now include this data in Fig. S6 and lines 279 – 285 in the "changes tracked" document.

Minor criticisms

1. The introduction could better reflect established FGF/ERK biology in neural patterning, given its pivotal role in MHB emergence

We thank the reviewer for the suggestion and now include a brief discussion of the important role of dynamic FGF/ERK signaling in midbrain-hindbrain boundary patterning in zebrafish:

© 2026. Published by The Company of Biologists under the terms of the Creative Commons Attribution License (https://creativecommons.org/licenses/by/4.0/).

Lines 49 – 50 in the "changes tracked" document: "*fgf8* ligand is expressed dynamically in the developing zebrafish brain (Moens and Prince, 2002) and contributes to the patterning activities of the midbrain-hindbrain boundary (Gibbs et al., 2017)."

2. The data suggest robust responsiveness from 30% epiboly onwards, while sphere-stage responses appear variable or not significant overall. The wording should reflect this distinction more precisely.

We have updated the text to clarify this possible point of confusion:

Line 274 – 275 in the "changes tracked" document: "Activation at sphere stage is therefore highly variable and not reliable in male-derived heterozygotes."

3. The analysis appears careful, but the manuscript would benefit from a clearer description of how ppERK signal was quantified and how spatial uniformity was assessed. In particular, the MA-IQR metric should be introduced in a way that is accessible to non-specialist readers.

We have updated both the main text and the methods section to more clearly explain how ppERK signal was quantified and the rationale and implementation of the MA-IQR measurements:

Line 209 – 212 in the "changes tracked" document: "Briefly, pixel intensities were arranged from lowest to highest and visualized as histograms. The difference between the 75th and 25th percentiles of this data was calculated as the IQR, a common measure of spread. To normalize for differences in absolute intensity between embryos, each embryo's IQR was then divided by its mean ppERK pixel intensity."

4. The finding that the fish can be maintained under the authors' standard facility lighting is useful. However, because lighting conditions vary between facilities, the conclusions and recommendations should remain appropriately cautious.

We agree with the reviewer that this finding should be appropriately caveated. We have updated the text as follows:

- Line 176 in the "changes tracked" document: "Under these conditions, *Tg(ubi:bOpto-FGF)* fish reliably breed." (Note: Removed "… do not require special light sources or husbandry considerations")
- Line 363 – 366 in the "changes tracked" document: "However, *Tg(ubi:bOpto-FGF)* fish survive and breed in our facility, even though our facility's white light sources contain emissions in the 420-525 nm range (Fig. 1E'). Importantly, we caveat that different lighting conditions could lead to problematic ectopic activation." (Note: This replaced a more speculative paragraph)

5. The possibility that this line may be useful beyond 24 hpf or in adults is interesting, but this remains speculative based on the present data. The manuscript should clearly separate demonstrated utility from future potential.

We have removed the line in the Discussion speculating that the tool may be usefully active beyond 24 hpf (formerly line 447-448, now removed from line 345 in "changes tracked" document).

This is a strong, well-executed and valuable resource paper. The manuscript is clearly suitable for Biology Open as a Methods & Techniques article. The main conclusions are supported, but several points require clarification, particularly the breadth of pathway validation, spatial activation claims, and interpretation of developmental consequences after activation.

<u>Reviewer 2:</u> This manuscript describes the generation and characterization of Tg(ubi:bOpto-FGF), a transgenic zebrafish line that ubiquitously expresses the bOpto-FGF optogenetic tool for blue light-inducible activation of FGF/ERK signaling. The authors demonstrate robust, spatially uniform ERK phosphorylation upon blue light exposure in both heterozygous and homozygous embryos, characterize light responsiveness across developmental stages from late blastula through 24 hpf,

© 2026. Published by The Company of Biologists under the terms of the Creative Commons Attribution License (https://creativecommons.org/licenses/by/4.0/).

and show that ectopic signaling is induced rapidly (within 3 minutes) and sustained for at least 75 minutes under continuous illumination. This is a useful methodological contribution to the zebrafish optogenetics toolkit.

Some points to consider for a revision:

The MATLAB-based quantification pipeline is referenced from a different paper (Iannucci et al., 2026), which is currently a preprint. The authors should either provide the code as supplementary material, deposit it in a public repository.

The referenced manuscript has recently been published in *Developmental Biology*: (Iannucci et al., 2026). We also now include a link to the MATLAB code directly in the current manuscript:
Lines 591 – 592 in the "changes tracked" document: "The MATLAB code can be accessed here: https://www.nichd.nih.gov/research/atNICHD/Investigators/rogers/software."

The claim of "ubiquitous" expression, central to the line's utility, is based on the known behavior of the ubi promoter as characterized in prior work (Mosimann et al., 2011). No direct evidence of uniform bOpto-FGF transgene expression across cell types is presented here, for example by anti-HA staining or fluorescent in situ for thetrangene. For a methods paper where this is a primary selling point, at minimum one representative image demonstrating broad expression would be expected including at later stages as this would increase the applicability of the method.

We appreciate this note from both reviewers and address it in the response to reviewer 1 above (Major Criticism #4).

The insertion site of the transgene is unknown. This ideally should be mapped in a primarily method paper presenting the transgenic line as main tool.

Our data are consistent with a reliable single insertion that segregates in a Mendelian fashion (heterozygous outcrosses result in ~50% of progeny receiving the transgene) and shows no evidence so far for silencing after several generations. Importantly, heterozygous embryos show robust light responses, and therefore if a user wants to cross the transgenic with a reporter line, there should be no issues even if the reporter and transgene happen to be on the same chromosome. Transgenics with unknown insertions are frequently used in the zebrafish community and we believe the *Tg(ubi:bOpto-FGF)* line could be used for many applications without identifying the insertion site. Together with the challenges associated with establishing a mapping pipeline *de novo* in our lab, we therefore feel that mapping the transgene insertion is outside the scope of the current manuscript.

To avoid possible activation of the construct by ambient light in fish raised for the line maintenance, the author should consider a bipartite system such as UAS/Gal4, Cre/Lox or QF/QUAS. This should be more clearly discussed in the manuscript.

We thank the reviewer for this elegant idea and have updated the Discussion to incorporate it:
Lines 368 – 370 in the "changes tracked" document: "Alternatively, a novel bipartite transgenic strategy could be designed using GAL4/UAS, Cre/Lox, or QF/QUAS to avoid potential challenges arising from light sensitive adults."

Minor Comments

1. Figure 4A legend notes that some time points were imaged with different settings. This aspect should also be noted in the main results text.

We have now added the following text to the main results:
Lines 247 – 250 in the "changes tracked" document: "3, 7, 10, and 39-minute timepoints were imaged on one day with the same imaging settings. 15, 63, and 79-minute timepoints were imaged the next day with different settings. These differences are accounted for by our fold change strategy."

© 2026. Published by The Company of Biologists under the terms of the Creative Commons Attribution License (https://creativecommons.org/licenses/by/4.0/).

2. The Iannucci et al., 2026 reference is currently a bioRxiv preprint. If it remains unpublished and not peer reviewed at the time of final submission, this should be noted, and key methodological details from that paper that are not reproduced here should be included.

The referenced manuscript has recently been published in *Developmental Biology*: (Iannucci et al., 2026).

References

Gibbs, H.C., Chang-Gonzalez, A., Hwang, W., Yeh, A.T., Lekven, A.C., 2017. Midbrain-Hindbrain Boundary Morphogenesis: At the Intersection of Wnt and Fgf Signaling. Front Neuroanat 11, 64.

Iannucci, L.E., Maria Thomas, V.S., Anderson, W.K., Murphy, M.R., Donahue, C.E.T., Campbell, C.E., Monaghan, M.T., Saul, A.J., Rogers, K.W., 2026. An optogenetic toolkit for robust activation of FGF, BMP, & Nodal signaling in zebrafish. Dev Biol 536, 155-171.

Kimmel, C.B., Ballard, W.W., Kimmel, S.R., Ullmann, B., Schilling, T.F., 1995. Stages of embryonic development of the zebrafish. Dev Dyn 203, 253-310.

Moens, C.B., Prince, V.E., 2002. Constructing the hindbrain: insights from the zebrafish. Dev Dyn 224, 1-17.

Second decision letter

MS ID#: bio.062695R1

MS Title: A transgenic zebrafish for spatiotemporal optogenetic activation of FGF/ERK signaling

Authors: William K. Anderson, Leanne E. Iannucci, Ninet Sinaii, Julia Porcino and Katherine W. Rogers

Thank you for submitting a revised manuscript and addressing all of the reviewer comments. After reading through your revised manuscript and responses to reviewer comments, I am happy to tell you that your manuscript has been accepted for publication in Biology Open, pending our standard publication integrity checks. It was accepted on 16th August 2026.

© 2026. Published by The Company of Biologists under the terms of the Creative Commons Attribution License (https://creativecommons.org/licenses/by/4.0/).
